# Impact of 4D‐CT MIP reconstruction method on iGTV definition: A clinical case report

**DOI:** 10.1002/acm2.70799

**Published:** 2026-09-17

**Authors:** Suhong Yu, Itai Pashtan

**Affiliations:** ^1^ Department of Radiation Oncology Mass General Brigham Cancer Institute Boston Massachusetts USA

**Keywords:** 4D‐CT, iGTV, maximum intensity projection, MIP reconstruction, quality assurance, respiratory motion management

## Abstract

To present a clinical case demonstrating the impact of 4D‐CT maximum intensity projection (MIP) reconstruction methodology on internal gross tumor volume (iGTV) definition and to highlight implications for motion management quality assurance. During routine thoracic radiation therapy treatment planning, a discrepancy in tumor extent was identified during physician contouring. The inferior extent of the tumor appeared artificially truncated on the MIP dataset used for target delineation. Further review revealed that the default MIP had been generated from phase‐sorted 4D‐CT images. A comparison was performed between the phase‐sorted MIP and a MIP reconstructed from the original cine images to evaluate differences in motion representation. The phase‐sorted MIP under‐represented the full tumor motion envelope, most notably in the inferior direction. In contrast, the cine‐based MIP demonstrated a more complete representation of tumor extent throughout respiration. This discrepancy was not readily apparent during the routine clinical workflow, and verification of the MIP reconstruction method was not part of the standard QA review process at the time. Differences in MIP reconstruction methodology can impact iGTV definition. Although these differences may be subtle in most cases, clinically meaningful discrepancies may occur, particularly in patients with irregular breathing patterns. This case highlights the importance of awareness and verification of 4D‐CT MIP reconstruction methods and supports comprehensive QA across CT simulation, treatment planning, and motion management workflows.

## 1 INTRODUCTION

Respiratory motion presents a major challenge in thoracic radiotherapy and can significantly impact target delineation and dose delivery. Multiple motion‐management strategies have been developed to address tumor motion, including deep inspiration breath hold (DIBH), respiratory gating, tumor tracking, and motion‐encompassing approaches using the internal gross tumor volume (iGTV).^1^ Each strategy relies on different assumptions regarding tumor motion and requires appropriate imaging and target definition techniques.

For respiratory‐gated treatments, phase‐sorted 4D‐CT images are typically used because radiation delivery is synchronized with a specific portion of the breathing cycle. In contrast, the motion‐encompassing iGTV approach aims to include the full range of tumor motion and is commonly used in lung SBRT.^1^, ^2^

Maximum intensity projection (MIP) images derived from 4D‐CT datasets are widely used to aid contouring iGTV, along with average intensity projection (Ave‐IP) images and review of individual respiratory phases.^5^, ^6^ MIP is particularly valuable for capturing the overall extent of tumor motion. However, accurate representation depends on proper reconstruction. Irregular breathing, phase‐sorting errors, and binning limitations may lead to incomplete representation of extreme tumor positions.^4^, ^7^

Prior studies have demonstrated that phase‐based MIP may underestimate motion compared to MIP reconstructed from original (cine) images.^3^, ^4^, ^7^ This report presents a clinical case highlighting this issue and its implications for quality assurance (QA) when establishing 4D‐CT motion management protocols.

## 2 CASE PRESENTATION

A discrepancy in target extent was identified by the treating physician during contouring, where the inferior portion of the tumor appeared artificially truncated on the MIP dataset.

**FIGURE 1:**
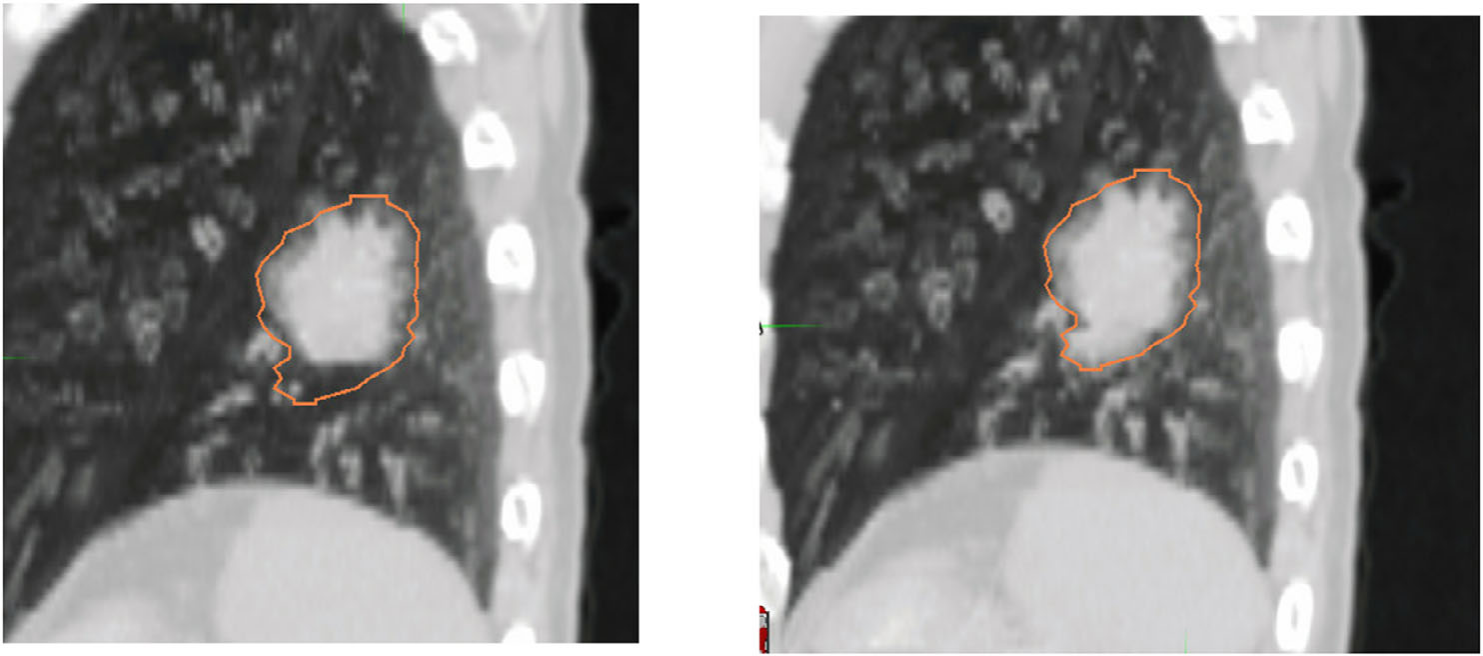
Comparison of MIP reconstruction methods (sagittal view). Left: MIP generated from phase‐sorted images showing truncated inferior tumor extent. Right: MIP generated from original (cine) images demonstrating a more complete motion envelope.

The scan was acquired on a GE CT scanner using a cine‐based deviceless 4D‐CT workflow. In this workflow, respiratory phase sorting is performed without an external respiratory surrogate, such as an RPM or RGSC marker block. Instead, the vendor software derives respiratory information from image‐based anatomic information within the cine CT acquisition.^8^, ^9^, ^10^ A user‐reviewable respiratory waveform derived from the 4D‐CT dataset was not available for review or export. The software also did not provide a user‐accessible image‐assignment log or a manual tool to reassign individual cine images to specific respiratory phases. The default clinical MIP was generated from the sorted phase images. For comparison, an additional MIP was generated in GE Advantage 4D using the vendor‐provided “Original Images” option within the MIP/Ave‐IP/Min‐IP reconstruction workflow. This option uses the available original cine images rather than only the sorted 10‐phase 4D‐CT image set. No in‐house reconstruction algorithm was used. The phase‐based MIP under‐represented the inferior tumor motion extent, whereas the original‐cine MIP demonstrated a more complete motion envelope (Figure 1).

**FIGURE 2:**
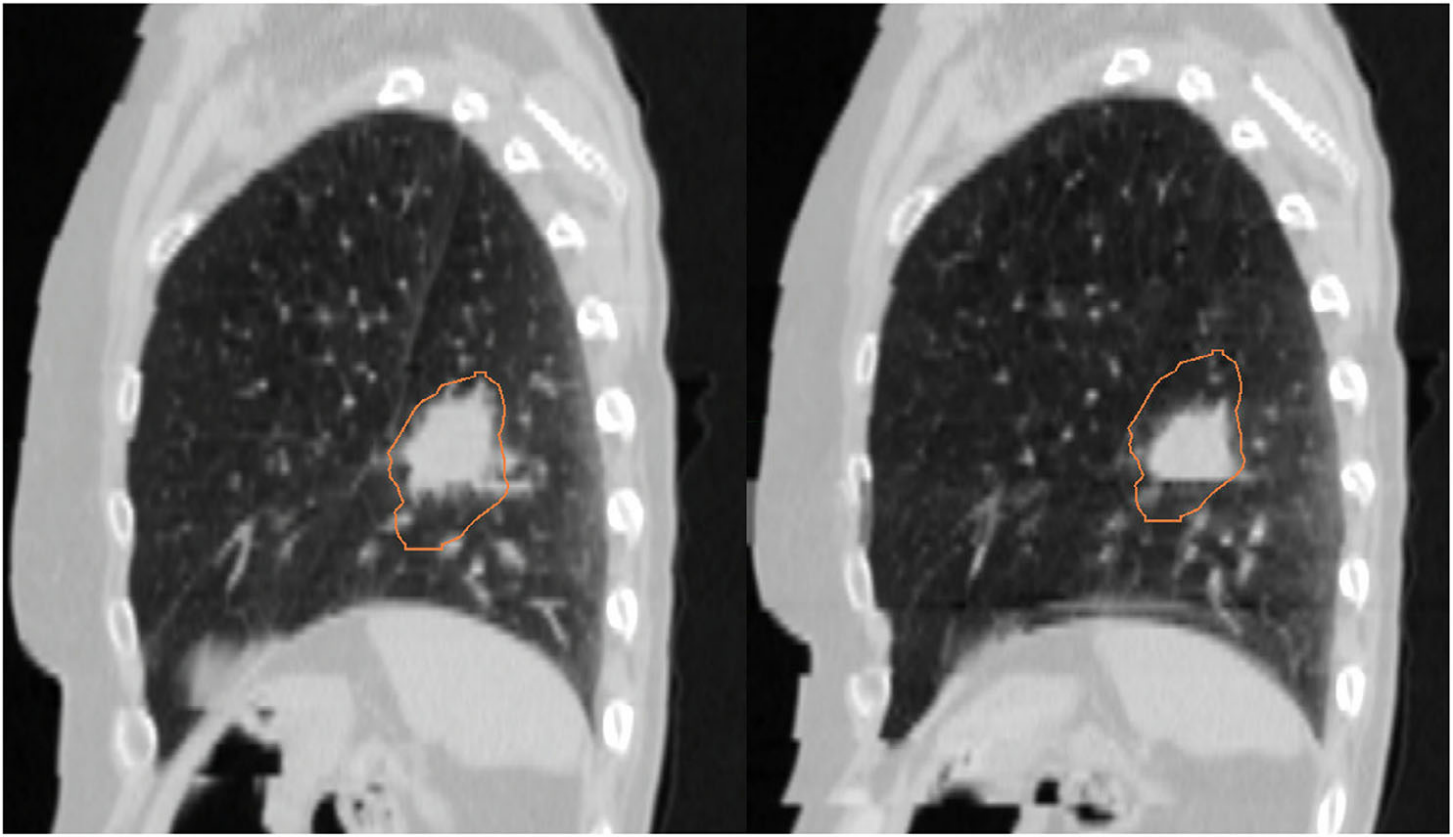
Representative 50% and 0% phase images. The inferior tumor extent is more completely visualized on the 50% phase image and appears truncated on the 0% phase image.

Because no external respiratory motion trace or user‐reviewable image‐derived waveform was available for review or export, the artifact was identified by reviewing the reconstructed 4D phase images. Representative 50% and 0% phase images were included to demonstrate the variable visualization of the inferior tumor extent across the sorted 4D dataset (Figure 2). The inferior tumor extent was more completely visualized on the 50% phase image and appeared truncated on the 0% phase image. This finding corresponded to the region where the phase‐based MIP underestimated the inferior tumor extent compared with the MIP reconstructed from the original cine image set in Figure 1.

This difference was not readily apparent in routine workflow.

## 3 DISCUSSION

Previous work by Zamora et al.^3^ demonstrated that phase‐based MIP reconstruction can underestimate tumor motion due to incomplete sampling and phase‐sorting limitations, particularly in the presence of irregular breathing. While this behavior is well described in the literature, it may not be routinely evaluated in clinical practice.

As illustrated in Figure 1, the phase‐based MIP demonstrates truncation of the inferior tumor extent, whereas the cine‐based reconstruction captures a more complete motion envelope.

The benefit of reconstructing a MIP from the original cine image set depends on whether the relevant tumor position was captured during image acquisition. A cine‐based MIP can only recover tumor extent that is present in the acquired cine images but not fully represented in the sorted phase image set. In this case, review of the reconstructed 4D phase images showed inconsistent visualization of the inferior tumor extent, with more complete visualization on the 50% phase image and apparent truncation on the 0% phase image. The MIP generated from the original cine images demonstrated a more complete inferior motion envelope. This suggests that the inferior tumor position was captured in the original cine acquisition but was under‐represented during respiratory sorting and phase‐based MIP generation. However, if the full tumor motion extent is not captured in the original acquisition, reconstruction from original cine images cannot recover the missing information, and repeat 4D‐CT simulation should be considered.

Prior phantom and clinical studies have described deviceless 4D‐CT as an internal‐anatomy‐based sorting approach and demonstrated general agreement with external‐surrogate‐based 4D‐CT.^8^, ^9^, ^10^ In a phantom‐based commissioning evaluation, Tillery et al.^8^ reported that GE Smart Deviceless 4D uses relationships between body contour and lung volume during reconstruction. Because deviceless sorting relies on internal anatomic motion rather than a single external surrogate signal, it may be more robust in some irregular breathing scenarios.^8^, ^10^

In this case, the original‐cine MIP demonstrated a more complete inferior tumor extent, indicating that the relevant tumor position was acquired but not fully represented in the sorted phase‐based MIP. However, because a user‐reviewable respiratory waveform and image‐assignment log were not available, the exact sorting behavior could not be directly verified.

For the majority of clinical cases, differences between reconstruction methods are often subtle. Routine contouring typically includes review of both MIP and all respiratory phases, which helps mitigate risk.

However, clinically meaningful discrepancies can occur, particularly in irregular breathing cases.^4^, ^7^ For ITV‐based approaches, accurate representation of full motion is critical; underestimation may lead to inadequate coverage.^1^, ^2^

The findings also emphasize the importance of understanding the reconstruction options available from different vendors when implementing or revising a clinical 4D‐CT motion‐management workflow. Awareness of the relevant literature and vendor‐specific functionality can help guide workflow selection and clinical review.

When the MIP demonstrates abrupt or nonphysiologic truncation, the reconstructed 4D phase images should be reviewed to assess whether the tumor extent is consistently represented throughout the respiratory cycle. A MIP generated from the original cine images may provide additional information when this option is available and the relevant tumor position was captured during acquisition. If the motion envelope remains uncertain, repeat 4D‐CT simulation should be considered. MIP images should be interpreted together with the individual phase images and clinical assessment rather than used as the sole basis for iGTV definition.

## 4 CONCLUSION

Differences in MIP reconstruction methodology can impact iGTV definition. Although these differences may be subtle in most cases, awareness and verification of the reconstruction approach are important to ensure accurate motion representation and support robust motion management workflows.

## AUTHOR CONTRIBUTIONS

All authors contributed to the conception, clinical review, manuscript preparation, and revision of this case report. All authors reviewed and approved the final manuscript.

## CONFLICT OF INTEREST STATEMENT

The authors declare no conflicts of interest.

## ETHICS STATEMENT

This work was reviewed by the Mass General Brigham Institutional Review Board and determined to be Not Human Subjects Research. Formal IRB approval and patient consent were not required. All data are fully de‐identified.

## Data Availability

The data supporting this case report is not publicly available because they contain clinical imaging data, even though all presented images and information have been de‐identified.
